# Early prediction of 3-month functional outcome after acute ischemic stroke: an explainable model based on routine clinical data

**DOI:** 10.3389/fneur.2026.1852875

**Published:** 2026-09-29

**Authors:** Zichen Rao, Chunyi Xu, Yanmei Yu, Yong Liu, Chunyan Zhu, Yiming Zhang

**Affiliations:** 1 Department of Endocrinology, Quzhou People’s Hospital, The Quzhou Affiliated Hospital, Wenzhou Medical University, Quzhou, Zhejiang, China; 2 Department of Endocrinology and Metabolism, Sir Run Run Shaw Hospital, Zhejiang University School of Medicine, Hangzhou, China; 3 Department of Rehabilitation Medicine, Zhejiang Provincial People's Hospital (Affiliated People's Hospital, Hangzhou Medical College), Center for Rehabilitation Medicine, Rehabilitation & Sports Medicine Research Institute of Zhejiang Province, Hangzhou, Zhejiang, China

**Keywords:** acute ischemic stroke, explainable models, functional outcome, machine learning, prognosis, risk stratification

## Abstract

**Background:**

Accurate early prediction of 3-month functional outcome after acute ischemic stroke is essential for prognostic communication, early management planning, and resource allocation. However, clinically applicable and interpretable prediction tools based on routinely collected data remain limited.

**Methods:**

In this single-center retrospective prediction-model study, we analyzed 770 adults with acute ischemic stroke admitted within 24 h of symptom onset. Baseline clinical and early laboratory variables were collected. After an 80/20 train–test split, key predictors were selected using cross-validated least absolute shrinkage and selection operator (LASSO) in the training set. Several supervised learning models were developed and compared. Model discrimination, calibration, and clinical utility were evaluated. The final gradient boosting machine (GBM) model was interpreted using TreeSHAP, and an online Shiny-based calculator was developed.

**Results:**

LASSO identified 12 predictors. Test-set discrimination was similar across models, with area under the receiver operating characteristic curve (AUC) values ranging from 0.854 to 0.867. The final GBM achieved AUCs of 0.910 in the training set and 0.872 in the test set, with corresponding areas under the precision–recall curve of 0.814 and 0.741. Calibration was satisfactory in both sets. SHAP analyses identified NIHSS, age, and C-reactive protein as dominant contributors, showing predominantly non-linear effects with limited interaction strength.

**Conclusion:**

An explainable model based on routinely collected clinical data showed good discrimination and calibration in internal validation for predicting 3-month functional outcome after AIS. Independent external validation is required before the model can be considered for clinical application.

## Introduction

Acute ischemic stroke (AIS) remains a leading cause of mortality and long-term disability worldwide (1, 2). Despite advances in acute reperfusion therapies and stroke-unit care, a substantial proportion of patients experience unfavorable functional outcomes, highlighting the need for accurate early prognostic assessment (3, 4). The 3-month modified Rankin Scale (mRS) is widely accepted as the standard measure of post-stroke functional outcome and is commonly used to guide clinical decision-making, rehabilitation planning, and prognostic communication (5, 6). However, predicting individual functional trajectories early after AIS remains challenging, owing to marked heterogeneity in stroke severity, vascular risk profiles, and systemic physiological responses.

Traditional regression-based prognostic models have long been used to estimate outcomes after acute ischemic stroke (7, 8). These models are usually built on a small number of prespecified variables and assume linear relationships between predictors and outcome. In routine clinical practice, however, stroke patients often differ widely in neurological severity, vascular risk burden, and systemic physiological status. Such heterogeneity makes it difficult for linear models to perform consistently across real-world populations, and their predictive accuracy is often reduced when applied outside selected cohorts (9, 10).

Machine-learning (ML) approaches provide a different way to address this problem. By allowing non-linear effects and interactions to emerge directly from the data, these models can better accommodate the complexity of stroke prognosis (11, 12). Several studies have reported improved performance of ML models in predicting functional outcome, hemorrhagic transformation, or mortality after stroke (8, 12, 13). Despite these encouraging results, clinical adoption has been limited. Many published models depend on large numbers of predictors that are not routinely available. Others apply complex preprocessing steps that are difficult to reproduce. Although explainability methods are increasingly used, differences in prediction targets, data requirements, validation strategies, and reporting continue to limit direct clinical translation (13–16).

In daily stroke care, clinicians rely on familiar indicators such as neurological deficit severity, metabolic status, inflammation, and organ function. Measures including the National Institutes of Health Stroke Scale (NIHSS), C-reactive protein (CRP), hemoglobin A1c (HbA1c), and renal function are all known to be associated with poor neurological recovery (17–20). What remains unclear is how these factors act together in individual patients. Their effects may not be linear, and their relative importance may vary across clinical contexts. Explainability methods, particularly SHapley Additive exPlanations (SHAP), make it possible to examine these patterns within complex models. By showing how each variable influences a given prediction, such approaches help bridge the gap between statistical performance and clinical understanding (21–23).

Previous explainable ML studies in stroke have addressed heterogeneous tasks, including stroke detection, recurrence, lesion location, and functional outcome, using data sources ranging from EEG signals and imaging to registry-based clinical variables (14–16, 21–26). Chen et al. included imaging and treatment variables in predicting 3-month functional outcome (26). In contrast, the present study used routine clinical, laboratory, and etiologic information without imaging-derived or treatment variables, and examined whether greater model complexity improved discrimination over logistic regression. Against this background, the present study focuses on a parsimonious model based on routine information available after the early admission assessment, with evaluation of discrimination, calibration, clinical net benefit, and patient-level interpretation for predicting 3-month functional outcome after AIS.

We therefore aimed to develop and internally evaluate an explainable model for predicting poor 3-month functional outcome after AIS using routinely collected information available after completion of the early admission assessment. Our goal was not only to achieve adequate predictive performance, but also to ensure that model outputs could be understood and evaluated in a clinical context. Accordingly, we applied structured feature selection, compared commonly used supervised learning approaches, and assessed discrimination, calibration, and clinical utility. To better understand how key clinical variables influenced individual predictions, we further examined model behavior using explainability analyses. The final model was designed to provide individualized risk estimates for research evaluation rather than to replace clinical judgment.

## Methods

### Study design and participants

Patient data meeting the eligibility criteria were retrospectively collected at Zhejiang Provincial People’s Hospital from September 2018 to September 2021. A total of 1,149 patients with acute ischemic stroke (AIS) admitted within 24 h of symptom onset were assessed for eligibility. AIS was diagnosed according to the World Health Organization criteria and confirmed by magnetic resonance imaging. Inclusion required age ≥18 years. Among the patients assessed, none were excluded for age <18 years, 36 were excluded for active infection at admission, 208 for unavailable 3-month mRS data, 54 for missing data in one or more required baseline predictors other than TOAST, and 81 for other prespecified exclusion criteria. Active infection at admission was excluded to minimize its potential influence on baseline inflammatory and laboratory measurements. The final study cohort comprised 770 patients. TOAST classification was unavailable for 93 patients. Missing TOAST values were not manually imputed and were handled according to the analysis stage, as described below. A schematic workflow of patient selection, data preprocessing, LASSO feature selection, model development, internal validation, explainability, and web-calculator deployment is shown in Figure 1.

**Figure 1:**
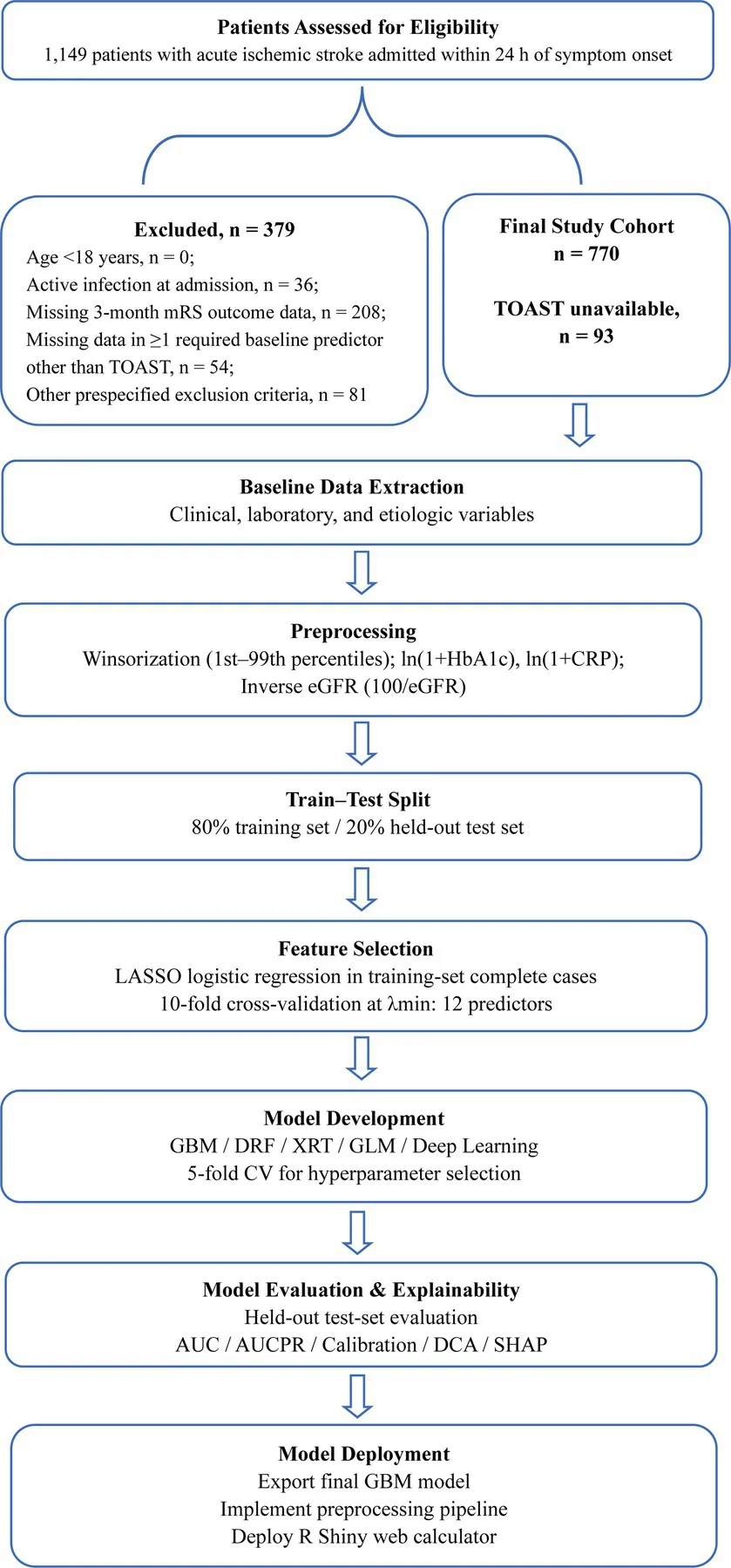
Workflow of patient enrollment, data preprocessing, feature selection, model development, internal evaluation, and deployment. A total of 770 patients with acute ischemic stroke (AIS) admitted within 24 h of onset were included. Baseline clinical, early laboratory, and etiologic variables were extracted. Preprocessing included winsorization and ln(1 + x) transformation for HbA1c and CRP, with renal function expressed as inverse eGFR (100/eGFR). Missing-data handling was analysis-specific: LASSO feature selection used complete cases for the candidate predictors, whereas downstream H2O models retained observations with missing TOAST classification using native missing-value handling. The dataset was split into an 80% training set and a 20% held-out test set before feature selection. LASSO logistic regression with 10-fold cross-validation at λ_min was applied to training-set complete cases, selecting 12 predictors. Four machine-learning models (GBM, DRF/XRT, GLM, and deep learning) were trained, and performance was assessed using AUC, AUCPR, decision-curve analysis (DCA), calibration, and SHAP-based explainability. The final GBM model was calibrated using 200 bootstrap resamples and deployed as a Shiny-based research calculator.

### Outcome assessment

Three-month functional outcome was assessed using the modified Rankin Scale (mRS) by neurologists trained in mRS assessment, through either face-to-face evaluation or telephone follow-up. Poor functional outcome was defined as mRS > 2. No specific blinding procedure was implemented for 3-month mRS assessment.

### Sample-size considerations

No formal *a priori* sample-size or statistical power calculation was performed because this retrospective study used all eligible patients available in the study database. Because the primary objective was prediction-model development rather than hypothesis testing, sample-size adequacy was considered in relation to the number of outcome events and candidate predictors. Among the 770 included patients, 206 experienced poor 3-month functional outcome and 564 did not. Because TOAST classification was missing in 93 patients, LASSO feature selection was performed using complete cases within the training set, without missing-value imputation at this stage; therefore, event-per-predictor ratios based on the full cohort were not reported. To reduce the risk of overfitting, feature selection and hyperparameter tuning were restricted to the training set, five-fold cross-validation was used during model development, and performance was subsequently evaluated in the held-out test set.

### Reporting guideline

This study was reported in accordance with the TRIPOD+AI statement for clinical prediction models developed using regression or machine-learning methods. A completed TRIPOD+AI checklist is provided as Supplementary file S1.

### Data collection and variable definitions

Baseline data were extracted from the hospital information system: age, sex, smoking status, systolic/diastolic blood pressure (SBP/DBP), and histories of hypertension, diabetes, atrial fibrillation (AF), and chronic obstructive pulmonary disease (COPD). Stroke severity and level of consciousness were assessed at admission using the National Institutes of Health Stroke Scale (NIHSS) and Glasgow Coma Scale (GCS), respectively. Swallowing function was evaluated using the Kubota Water Drinking Test (KWDT), and the A2DS2 score was calculated from admission data to characterize the subsequent risk of stroke-associated pneumonia. A2DS2 was reported descriptively and was not included in the candidate predictor set. Blood samples obtained at hospital admission were used for routine biochemistry and hematology, including triglycerides (TG), LDL-C, HDL-C, total cholesterol, C-reactive protein (CRP), hemoglobin A1c (HbA1c), blood urea nitrogen (BUN), liver enzymes (ALT/AST), homocysteine (HCY), uric acid (UA), white blood cell count (WBC), hemoglobin, vitamin B12, free triiodothyronine (FT3), free thyroxine (FT4), and serum creatinine for calculation of eGFR using the CKD-EPI equation. The FT3/FT4 ratio was calculated by dividing FT3 by FT4. Fasting plasma glucose (FPG) was measured on the morning of hospital day 2 and was retained only for descriptive characterization of the cohort; it was not considered an admission predictor.

To address skewed distributions, HbA1c and CRP underwent 1st–99th percentile winsorization followed by ln(1 + x) transformation. Renal function was represented as inverse eGFR (100/eGFR). Stroke etiology was classified according to the Trial of Org 10172 in Acute Stroke Treatment (TOAST) criteria. For model development, TOAST subtype was dichotomized as large-artery atherosclerosis (LAA) versus non-LAA. LAA was coded as 1, and all other TOAST subtypes were coded as 0. TOAST classification was unavailable for 93 patients; these missing values remained coded as missing and were not assigned to the non-LAA category.

### Feature selection

Candidate predictors were defined before model fitting based on their availability by completion of the routine early admission assessment, their clinical relevance to 3-month functional outcome after AIS, and the avoidance of redundant representation of the same clinical construct. The candidate pool comprised age, sex, smoking status, hypertension, SBP, diabetes, AF, COPD, TOAST subtype (LAA vs. non-LAA), HbA1c, CRP, inverse eGFR, TG, HDL-C, LDL-C, AST, UA, HCY, FT3/FT4 ratio, vitamin B12, NIHSS, and KWDT. Variables shown in Table 1 but not entered into LASSO—including DBP, FPG, total cholesterol, ALT, BUN, WBC, hemoglobin, GCS, and A2DS2—were included only to provide a broader description of the study cohort. FPG was not included in the candidate predictor set because it was measured on hospital day 2 and fell outside the predefined prediction window; therefore, it was not used in LASSO or any downstream model. GCS and A2DS2 were excluded to reduce redundancy with NIHSS and other included clinical variables. Feature selection was performed using LASSO logistic regression implemented in EmpowerStats (AutoLASSO module) with 10-fold cross-validation. To avoid information leakage, the dataset was first split into an 80% training set and a 20% held-out test set; LASSO fitting and cross-validation were conducted exclusively within the training set. For LASSO feature selection, complete-case analysis was used within the training set; observations with missing candidate predictors were excluded, and no imputation was performed at this stage.

**Table 1:** Baseline characteristics of the study cohort stratified by 3-month functional outcome (mRS ≤ 2 vs. > 2).

Characteristic	3 months mRS ≤ 2	3 months mRS > 2	*p*-value
*N*	564	206	
Age (years)	67.79 ± 11.68	73.20 ± 12.98	<0.001
SBP (mmHg)	153.00 (138.00–169.00)	155.50 (140.00–170.00)	0.152
DBP (mmHg)	83.00 (74.75–90.00)	82.50 (73.00–91.00)	0.737
FPG (mmol/L)	6.32 ± 2.45	6.97 ± 3.52	<0.001
TG (mmol/L)	1.31 (0.91–1.88)	1.10 (0.80–1.55)	<0.001
HbA1C (%)	6.59 ± 1.68	6.71 ± 1.77	0.317
TC (mmol/L)	4.28 (3.60–5.01)	4.40 (3.70–5.22)	0.317
HDL-C (mmol/L)	1.13 (0.97–1.33)	1.19 (1.00–1.38)	0.104
LDL-C (mmol/L)	2.71 (2.14–3.40)	2.87 (2.17–3.64)	0.206
CRP (mg/L)	2.55 (1.21–5.49)	6.13 (2.05–20.75)	<0.001
AST (U/L)	19.00 (16.00–24.00)	20.20 (17.00–26.00)	0.009
ALT (U/L)	18.00 (13.00–27.00)	16.00 (12.00–23.75)	0.046
BUN (mmol/L)	5.48 ± 2.22	6.04 ± 3.20	0.109
UA (μmol/L)	328.96 ± 90.14	326.33 ± 107.72	0.448
HCY (μmol/L)	15.10 (11.74–19.07)	16.55 (13.12–20.17)	0.004
FT3/FT4 ratio	0.30 (0.26–0.34)	0.26 (0.23–0.29)	<0.001
WBC (×10^9^/L)	7.17 ± 2.55	8.50 ± 3.69	<0.001
Vitamin B12 (pg/mL)	340.50 (249.75–482.00)	343.50 (233.00–490.75)	0.818
Hemoglobin (g/L)	135.00 (126.00–147.00)	132.00 (119.00–143.00)	0.002
eGFR (mL/min/1.73 m^2^)	103.65 ± 35.81	98.75 ± 42.04	0.071
NIHSS score	2.00 (1.00–4.00)	6.00 (3.00–10.00)	<0.001
KWDT score	1.00 (1.00–1.00)	1.00 (1.00–3.00)	<0.001
GCS score	15.00 (14.00–15.00)	13.00 (11.00–14.00)	<0.001
A2DS2 score	2.00 (2.00–3.00)	4.00 (3.00–5.00)	<0.001
Sex (*n*, %)			0.005
Female	221 (39.18%)	104 (50.49%)	
Male	343 (60.82%)	102 (49.51%)	
Smoking (*n*, %)			0.004
No	356 (63.12%)	153 (74.27%)	
Yes	208 (36.88%)	53 (25.73%)	
Hypertension			0.204
No	131 (23.23%)	57 (27.67%)	
Yes	433 (76.77%)	149 (72.33%)	
Diabetes (*n*, %)			0.019
No	380 (67.38%)	120 (58.25%)	
Yes	184 (32.62%)	86 (41.75%)	
Atrial fibrillation (*n*, %)			<0.001
No	493 (87.41%)	143 (69.42%)	
Yes	71 (12.59%)	63 (30.58%)	
COPD			0.007
No	532 (94.33%)	183 (88.83%)	
Yes	32 (5.67%)	23 (11.17%)	
TOAST			0.223
LAA	263 (50.00%)	84 (55.63%)	
No-LAA	263 (50.00%)	67 (44.37%)	

Data are summarized as mean ± SD or median (IQR), and categorical variables as *n* (%). TOAST classification was available for 526 patients in the mRS ≤2 group and 151 patients in the mRS >2 group; percentages for TOAST were calculated using the available observations within each outcome group. TOAST data were missing for 38 and 55 patients, respectively. Two-sided tests were used throughout: Student’s *t*-test for normally distributed continuous variables, Mann–Whitney *U* test for non-normal distributions, and *χ*^2^ test or Fisher’s exact test for categorical variables, as appropriate. Because the between-group comparisons in this table were descriptive and exploratory, no adjustment for multiple comparisons was applied. The reported p values should therefore be interpreted descriptively rather than as confirmatory evidence. Three-month functional outcome was classified by the modified Rankin Scale (mRS): mRS ≤ 2 vs mRS > 2.

mRS, modified Rankin Scale; SBP/DBP, systolic/diastolic blood pressure; FPG, fasting plasma glucose; TG, triglycerides; TC, total cholesterol; HDL/LDL, high-/low-density lipoprotein cholesterol; CRP, C-reactive protein; AST/ALT, aspartate/alanine aminotransferase; BUN, blood urea nitrogen; UA, uric acid; HCY, homocysteine; WBC, white blood cell count; eGFR, estimated glomerular filtration rate; NIHSS, National Institutes of Health Stroke Scale; GCS, Glasgow Coma Scale; A2DS2, score for post-stroke aspiration pneumonia risk; KWDT, Kubota Water Drinking Test; A2DS2, a clinical score for predicting stroke-associated pneumonia; FT3, free triiodothyronine; FT4, free thyroxine.

Not all variables shown in this table were entered into LASSO; the candidate predictor set is specified in the Methods.

Preprocessing steps applied in EmpowerStats (winsorization, ln-transforms, inverse eGFR) were replicated identically in R. No additional scaling or normalization was applied.

Cross-validation identified λ_min as the optimal penalty, yielding a clinically interpretable set of 12 predictors: Age, Sex, Smoking, SBP, Diabetes, AF, LDL-C, TOAST subtype, ln(1 + HbA1c), ln(1 + CRP), inverse eGFR, and NIHSS. TOAST subtype was entered as a single binary predictor, with LAA coded as 1 and all other TOAST subtypes grouped as non-LAA and coded as 0. Results for λ_1se were computed but not used for downstream modeling because λ_min showed higher observed discrimination while retaining a clinically interpretable predictor set.

Internal calibration in EmpowerStats used 200 bootstrap iterations, reporting apparent and optimism-corrected Brier score, calibration slope, and intercept.

### Model development and internal validation

After LASSO selection, the 12 retained predictors were used for downstream model development. The 80/20 train-test split used for LASSO was preserved for model training and evaluation. The preserved split comprised 616 patients in the training set and 154 patients in the held-out test set. The test set included 40 patients with poor 3-month functional outcome and 114 with favorable outcome. The held-out test set was not used for feature selection, model fitting, or hyperparameter selection. All performance metrics reported in Table 2, as well as the test-set metrics shown in Figure 2, were calculated exclusively from these 154 patients. Because the held-out test set was derived from the same single-center cohort, it constituted split-sample internal validation rather than external validation. No independent external validation cohort was available for the present study. All preprocessing transformations were fit using the training set only and applied unchanged to the test set to prevent leakage.

**Table 2:** Comparison of different machine learning algorithms for predicting 3-month functional outcomes after stroke.

Models	AUC	Sensitivity	Specificity	PPV	NPV	Accuracy
Gradient boosting machine (GBM)	0.867 (0.834, 0.900)	0.825 (0.770, 0.872)	0.853 (0.822, 0.880)	0.674 (0.615, 0.729)	0.930 (0.906, 0.950)	0.846 (0.819, 0.870)
Random forest (DRF/XRT)	0.854 (0.820, 0.888)	0.775 (0.713, 0.826)	0.862 (0.832, 0.888)	0.673 (0.612, 0.730)	0.913 (0.885, 0.933)	0.839 (0.811, 0.863)
Generalized linear model (GLM)	0.859 (0.825, 0.893)	0.825 (0.770, 0.872)	0.784 (0.749, 0.817)	0.584 (0.528, 0.640)	0.924 (0.898, 0.945)	0.795 (0.766, 0.823)
Deep learning (DL)	0.855 (0.821, 0.889)	0.850 (0.796, 0.893)	0.810 (0.775, 0.840)	0.622 (0.563, 0.675)	0.936 (0.912, 0.955)	0.821 (0.792, 0.846)

AUC, area under the receiver operating characteristic curve; PPV, positive predictive value; NPV, negative predictive value.

Initial model training and hyperparameter selection were performed using the H2O AutoML backend through R code generated by EmpowerStats. Hyperparameter tuning was conducted within the 80% training set using five-fold cross-validation. AutoML was run with a maximum of 20 candidate models and a fixed random seed of 1,234. No manually specified hyperparameter grid was used; algorithm-specific hyperparameters were selected automatically according to the default procedures of H2O AutoML. The 20% held-out test set was reserved for subsequent performance evaluation and was not used during hyperparameter tuning. DRF and XRT were evaluated as separate variants within the distributed random forest family; “DRF/XRT” is used as a family-level label in this table. Test-set discrimination was similar across the candidate models. GBM was selected as the final model for subsequent explainability analysis and web-calculator development based on its favorable overall discrimination and calibration profile and its compatibility with TreeSHAP-based interpretation, rather than on evidence of statistically superior discrimination.

**Figure 2:**
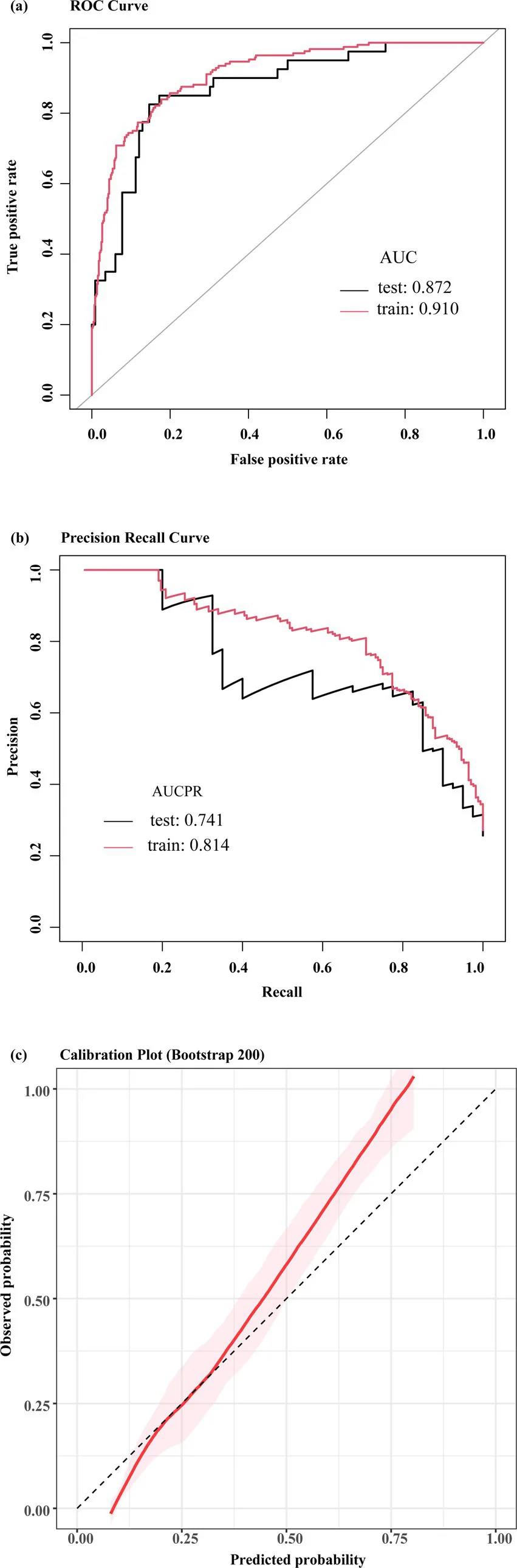
Internal performance of the final GBM model. **(a)** ROC curves for the GBM model in the training and held-out test sets (AUC 0.910 vs. 0.872).**(b)** Precision–recall curves for the training and held-out test sets (AUCPR 0.814 vs. 0.741). **(c)** Calibration plot based on 200 bootstrap resamples, showing predicted versus observed probabilities. The diagonal dashed line indicates perfect calibration, and the shaded region reflects bootstrap-derived uncertainty around the calibration curve.

Initial model training and algorithm-specific hyperparameter selection were conducted through the AutoML module in EmpowerStats, which generated R code and used the H2O AutoML backend. Hyperparameter tuning was performed exclusively within the training set using five-fold cross-validation. No manually specified hyperparameter grid was imposed; instead, algorithm-specific hyperparameter combinations were explored and selected automatically through the H2O AutoML procedure. The held-out test set was reserved for subsequent performance evaluation and was not used during hyperparameter tuning. For downstream H2O AutoML model development, patients with missing TOAST classification were retained. Missing TOAST values were handled using the algorithm-specific native missing-value mechanisms in H2O, and no manual imputation was performed.

Four algorithm families were evaluated:

(1) gradient boosting machine (GBM),
(2) the distributed random forest family,
(3) generalized linear model with logistic regression (GLM), and
(4) feed-forward deep learning.

Within the distributed random forest family, distributed random forest (DRF) and extremely randomized trees (XRT) were evaluated as separate model variants. Accordingly, the notation “DRF/XRT” refers to the distributed random forest family rather than to a single hybrid algorithm. The selected models were subsequently implemented in R using the same predictors and data split to support reproducibility and to enable downstream performance evaluation, explainability analysis, and web-calculator deployment.

Five-fold cross-validation within the training set was used for algorithm-specific hyperparameter selection and model development. Final model performance was subsequently evaluated on the held-out test set, which was not used during model tuning. Because discrimination was similar across the candidate models, the GBM was selected as the final model based on its favorable overall discrimination and calibration profile, together with its compatibility with TreeSHAP-based interpretation and subsequent web-calculator deployment, rather than on evidence of statistically superior discrimination.

Calibration of the final GBM was additionally evaluated in R using bootstrapped calibration curves and calibration-slope/intercept estimates.

### Performance metrics and operating points

Primary discrimination was assessed using the area under the ROC curve (AUC) with 95% confidence intervals estimated by DeLong’s method. The area under the precision–recall curve (AUCPR) was also reported. Between-model differences in AUC were interpreted descriptively and were not considered evidence of statistically superior discrimination. Operating characteristics—including sensitivity, specificity, positive predictive value (PPV), negative predictive value (NPV), and accuracy—were evaluated at the Youden-optimal probability threshold. Wilson 95% CIs were used for proportion-based metrics.

### Decision-curve analysis

To assess clinical utility, we performed decision-curve analysis (DCA) on the test set using R, plotting the net benefit of the final GBM model across threshold probabilities and comparing it with treat-all and treat-none strategies.

### Explainability

Model explainability was performed entirely in R using TreeSHAP applied to the final GBM model (rebuilt in R). We generated:

(i) global feature importance based on mean absolute SHAP values;
(ii) SHAP summary (beeswarm) plots to visualize distribution and directional effects;
(iii) SHAP dependence plots to evaluate non-linear and interaction-like behavior; and
(iv) pairwise interaction heatmaps using Friedman’s H-statistic to quantify potential effect modification.

### Software and web calculator

LASSO and AutoML model development were executed in EmpowerStats (version 5.2) with embedded R 4.2. The AutoML module generated R code and used the H2O AutoML backend (version 3.44.0.3) for model training and algorithm-specific hyperparameter selection. All additional analyses—including model refitting, ROC analysis, DeLong CIs, DCA and SHAP explainability—were performed in R 4.2 using packages including pROC (version 1.18.5), rmda (version 1.6), glmnet (version 4.1–8), and ggplot2 (version 3.5.1).

The final GBM model implemented in R was exported and deployed as an online risk-prediction calculator using the Shiny package (version 1.8.1.1). The calculator (https://stroke-ai-gbm.shinyapps.io/rrr_8_tbl/) provides real-time estimation of the probability of poor 3-month functional outcome (mRS > 2) based on the 12-predictor GBM model.

## Results

### Baseline characteristics by 3-month functional outcome (mRS ≤ 2 vs. > 2)

Patients with poor outcome were older (*p* < 0.001) and had higher fasting glucose, CRP, AST, ALT, homocysteine, WBC, and NIHSS, as well as higher KWDT and A2DS2 scores (all *p* < 0.01). Triglycerides and hemoglobin were lower (both *p* < 0.01). The poor-outcome group included more women, fewer current smokers, and higher proportions of diabetes, atrial fibrillation, and COPD (all *p* < 0.05). Systolic/diastolic blood pressure, total cholesterol, HDL-C, LDL-C, BUN, uric acid, vitamin B12, eGFR showed no statistically significant differences between groups (all *p* > 0.05). Among patients with available TOAST classification, the distribution of LAA versus non-LAA also did not differ significantly between outcome groups (*p* = 0.223) (Table 1). The identical LAA and non-LAA counts in the favorable-outcome group (263 vs. 263) reflected the observed distribution among patients with available TOAST classification.

### LASSO-derived predictive signature at λ_min

Cross-validated LASSO selected λ_min (Figure 3a). Coefficient paths decreased with increasing λ (Figure 3b). At λ_min, 12 predictors had non-zero coefficients: Age, Sex, Smoking, SBP, Diabetes, AF, LDL-C, TOAST classification (LAA vs. non-LAA), HbA1c (ln[1 + HbA1c]), CRP (ln[1 + CRP]), inverse eGFR (100/eGFR), and NIHSS. The λ_min model had AUC = 0.859 (Figure 3c). After 200-bootstrap internal validation, apparent Brier score was 0.12, optimism-corrected Brier 0.13, calibration intercept 0.04, and slope 1.04 (Figure 3d).

**Figure 3:**
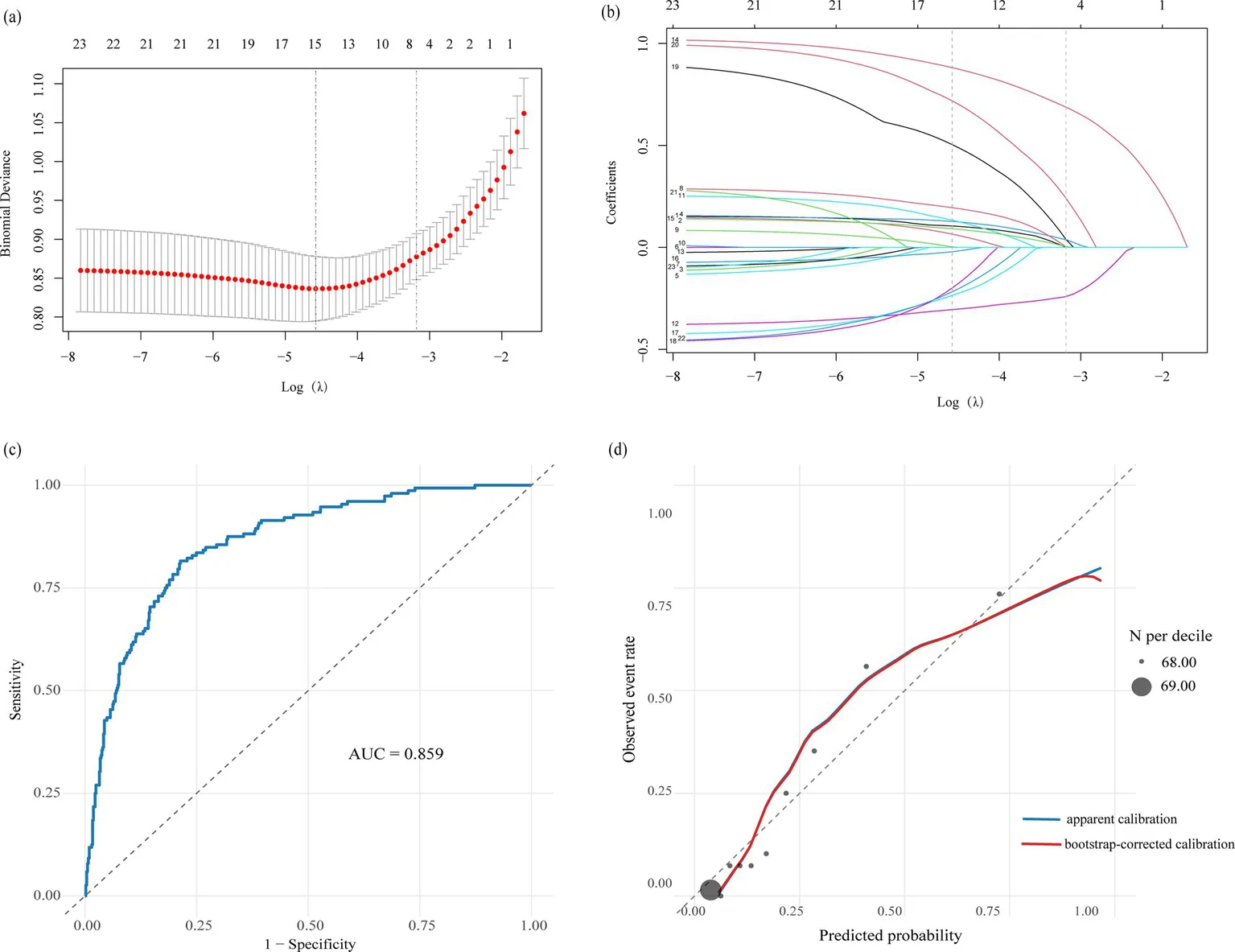
Development and internal validation of the LASSO prediction model. **(a)** Ten-fold cross-validated binomial deviance curve for LASSO logistic regression. Red dots represent mean deviance with ±1 SE; vertical dashed lines indicate λ_min and λ_1se, and the numbers along the upper axis indicate the number of non-zero coefficients. **(b)** LASSO coefficient paths as a function of log(λ), with vertical dashed lines marking λ_min and λ_1se. **(c)** ROC curve of the λ_min model in the held-out test set (AUC = 0.859). **(d)** Calibration plot based on 200 bootstrap resamples. Gray points represent observed event rates by risk deciles, the blue and red curves represent apparent and bootstrap-corrected calibration, respectively, and the diagonal dashed line indicates perfect calibration. The optimism-corrected Brier score was 0.132.

### Model comparison for 3-month outcome prediction

Using the 12 predictors, four classifiers were trained: gradient boosting (GBM), random forest (DRF/XRT), generalized linear model (logistic regression), and a simple deep-learning model. Five-fold cross-validation was used within the training set for hyperparameter selection, whereas the performance estimates reported below were obtained from the held-out test set. Test-set ROC curves are shown in Figure 4a. AUCs with 95% CIs were: GBM 0.867 (0.834–0.900), GLM 0.859 (0.825–0.893), DL 0.855 (0.821–0.889), and RF 0.854 (0.820–0.888) (Table 2). Thus, test-set discrimination was closely similar across the four algorithms. Although GBM had the numerically highest AUC, the small numerical differences were not interpreted as evidence of statistically superior discrimination. At the Youden-optimal threshold, GBM yielded Sensitivity 0.825 (0.770–0.872), Specificity 0.853 (0.822–0.880), PPV 0.674 (0.615–0.729), NPV 0.930 (0.906–0.950), Accuracy 0.846 (0.819–0.870) (Table 2). Decision-curve analysis indicated net benefit above the “treat-none” strategy across a range of thresholds for all models (Figure 4b). Calibration curves on the testing set (Figure 4c) indicated that GBM and GLM predictions were positioned near the 45° reference line across much of the probability range; the deep-learning model displayed mild deviations from this pattern; and the random-forest model exhibited higher observed risks than predicted in several intermediate-to-high probability regions. GBM was used for subsequent SHAP analysis.

**Figure 4:**
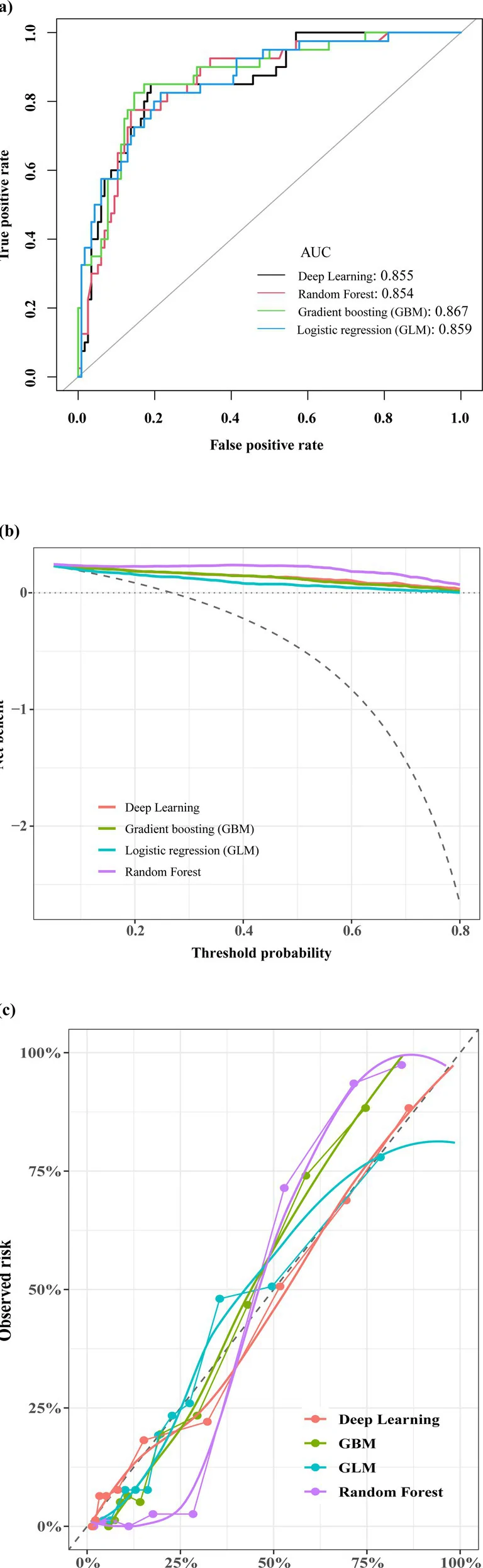
Comparison of machine-learning models in the held-out test set. **(a)** ROC curves for four algorithms. Test-set AUCs were 0.855 for deep learning, 0.854 for random forest, 0.867 for GBM, and 0.859 for GLM. **(b)** Decision-curve analysis comparing net benefit across threshold probabilities; the reference lines represent the treat-all and treat-none strategies. **(c)** Calibration curves for the four algorithms. The diagonal reference line represents perfect calibration. GBM and GLM were relatively close to this reference line, deep learning showed mild deviation, and random forest showed higher observed risks than predicted in several intermediate-to-high probability regions.

### Split-sample internal validation of the final GBM model

With an 80/20 train–test split, AUCs were 0.910 (training) and 0.872 (testing) (Figure 2a). AUCPR values were 0.814 (training) and 0.741 (testing) (Figure 2b). Calibration assessment using 200-bootstrap resampling showed that predicted probabilities were close to observed risks across the probability range, with the calibration curve lying near the 45° reference line and displaying mild deviation (Figure 2c). These findings describe the discrimination and calibration performance of the final GBM model. Both AUC and AUCPR were lower in the held-out test set than in the training set, indicating some loss of predictive performance on unseen data.

### SHAP-based interpretation of the final GBM model

On the testing set, SHAP global importance ranked variables as follows (Figure 5a): NIHSS, Age, CRP, TOAST classification (LAA vs. non-LAA), AF, eGFR, LDL-C, Diabetes, SBP, Smoking, Sex, HbA1c. The beeswarm plot displays the direction of SHAP contributions (Figure 5b). SHAP dependence plots showed non-linear associations (Figures 6a–c): NIHSS-related SHAP values increased sharply from approximately 4–6 and continued to rise across higher NIHSS values, reaching a high level around 10–15. CRP contributions increased at lower values and appeared to level off at higher values; and Age contributions rose more sharply at higher ages. LDL-C dependence varied across Smoking and eGFR strata (Figures 6d–e). The Friedman H-statistic heatmap showed low-to-moderate interaction strengths, with relatively higher values for eGFR×LDL-C and LDL-C × Smoking (Figure 6f).

**Figure 5:**
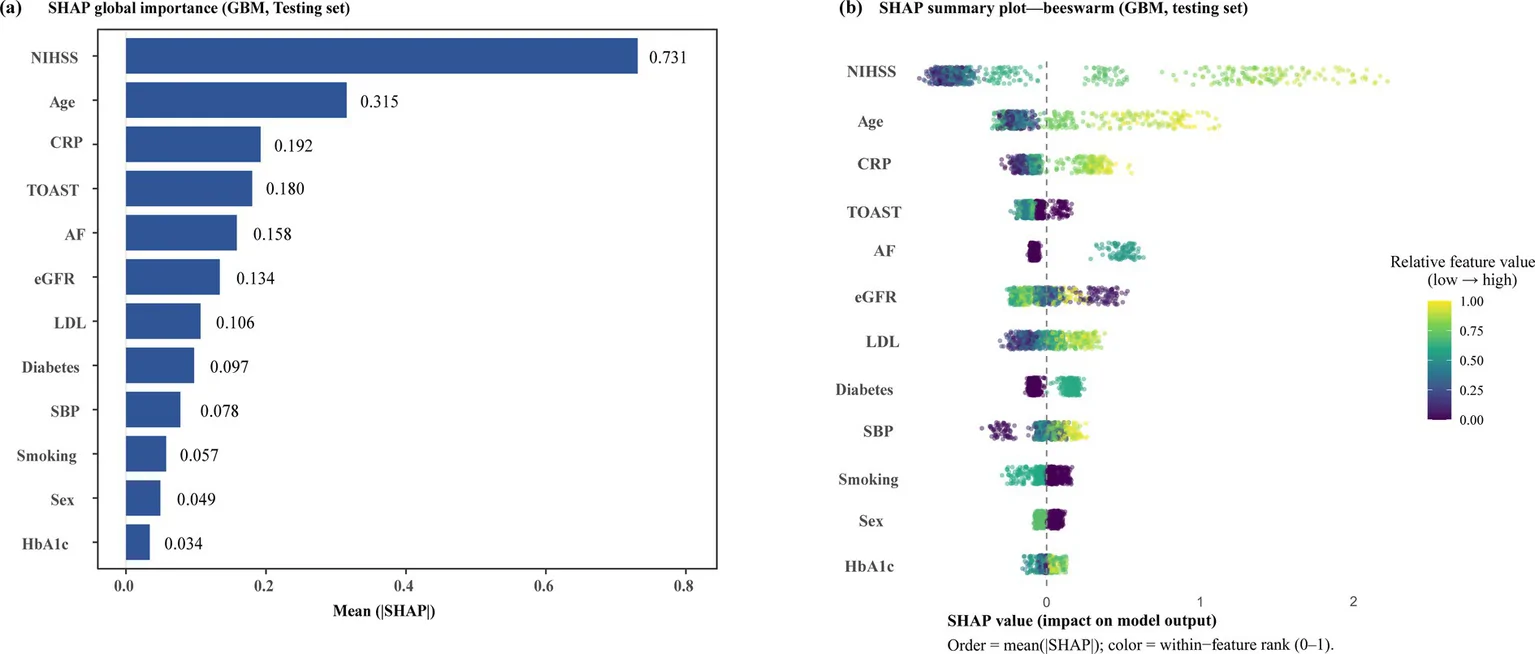
Model interpretability with SHAP: global importance and beeswarm summary. **(a)** Global feature importance ranked by mean absolute SHAP value, with larger values indicating greater overall contributions to model predictions. **(b)** Beeswarm summary displaying patient-level SHAP distributions. Each point represents an individual patient, and color indicates the relative within-feature value from low to high. Positive SHAP values indicate contributions toward a higher predicted probability of poor 3-month functional outcome, whereas negative values indicate contributions toward a lower predicted probability.

**Figure 6:**
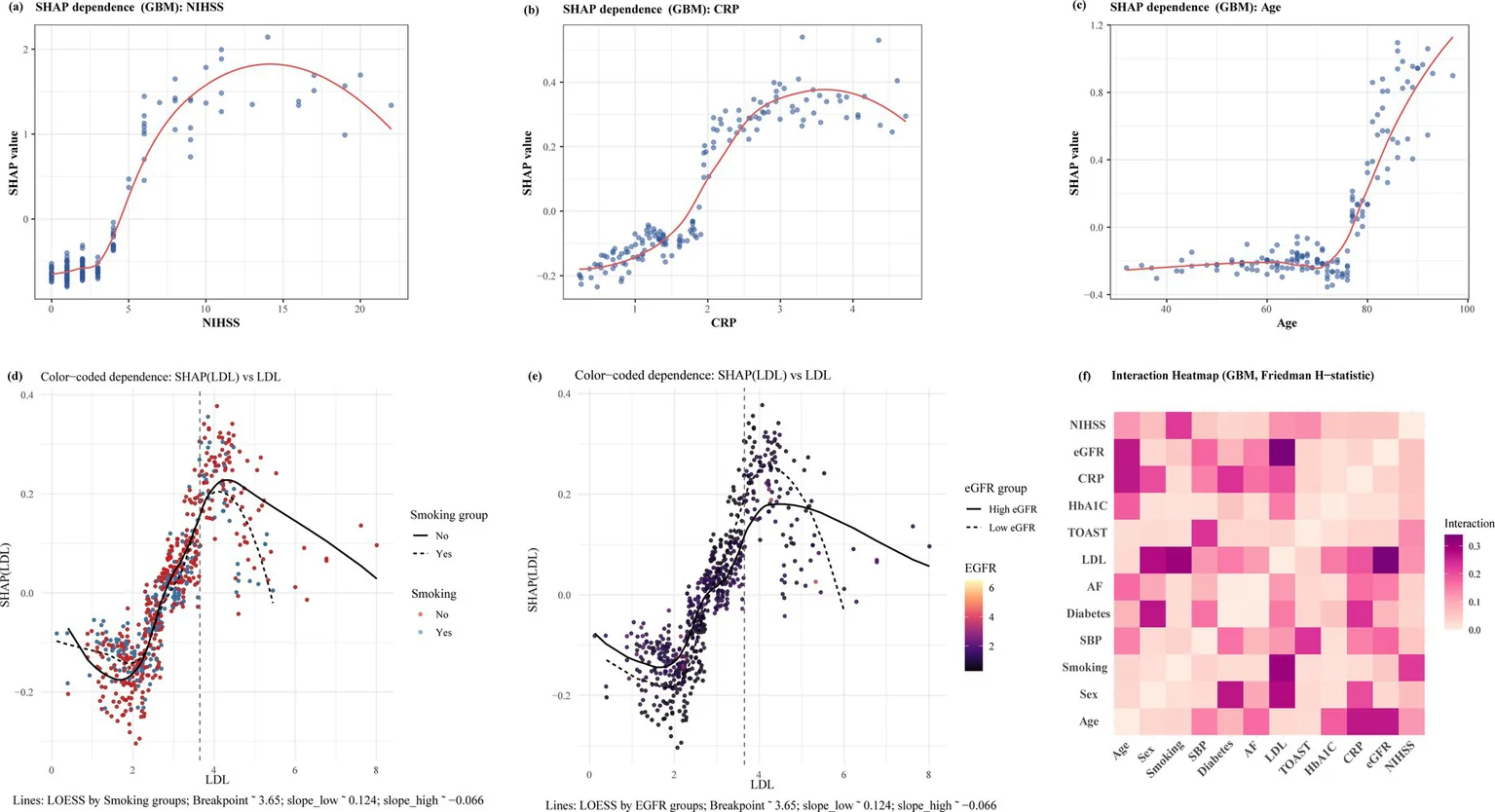
SHAP interpretation of marginal effects and pairwise interactions. **(a–c)** SHAP dependence plots for NIHSS, CRP, and age, respectively. Each point represents an individual observation, and the smoothed curve illustrates the non-linear pattern between the predictor value and its SHAP contribution. **(d)** LDL-C dependence according to smoking status, with points and fitted curves distinguishing smoking groups. The vertical dashed line marks the reference breakpoint at approximately 3.65. **(e)** LDL-C dependence according to eGFR, with point color reflecting the eGFR-related value and fitted curves shown for the high- and low-eGFR groups. The vertical dashed line marks the same reference breakpoint. **(f)** Friedman H-statistic interaction heatmap showing pairwise interaction strength; larger H-statistic values indicate stronger interactions, with relatively higher values observed for eGFR×LDL-C and LDL-C × Smoking.

### Deployment of an interactive web-based risk calculator

To enhance clinical accessibility and facilitate individualized risk estimation, the final GBM model was deployed as an online interactive calculator using the R Shiny framework. The calculator allows users to input the 12 predictors selected by LASSO and returns the predicted probability of poor 3-month functional outcome (mRS > 2). The tool incorporates the same preprocessing pipeline and final GBM model used in the present study, ensuring consistency between the web interface and the original model predictions. The calculator is provided as a research prototype and is not intended for clinical decision-making before independent external validation. The application was deployed on the Shinyapps.io server and is accessible at: https://stroke-ai-gbm.shinyapps.io/rrr_8_tbl/.

Internal testing confirmed that predictions generated by the web calculator matched those from the GBM model rebuilt in R. The interface displays both processed inputs and predicted probabilities.

## Discussion

In this cohort of 770 patients with acute ischemic stroke, we examined early predictors of 3-month functional outcome using routinely available clinical data. Several factors consistently contributed to outcome prediction, with neurological deficit severity, age, inflammatory status, and renal function emerging as the most influential variables. These factors showed predominantly non-linear effects, underscoring the heterogeneity of recovery trajectories after stroke. Importantly, their contributions varied across individuals, highlighting that patients with similar clinical profiles may carry different levels of functional risk. Together, these findings suggest that early outcome assessment after acute ischemic stroke may benefit from approaches that capture individual-level variability rather than relying solely on uniform prognostic assumptions.

Discrimination was similar across the four candidate algorithms in the held-out test set, with AUCs ranging from 0.854 to 0.867. Thus, the present findings do not demonstrate that GBM outperformed logistic regression or the other candidate algorithms. This observation is consistent with previous evidence that increasing model complexity does not necessarily translate into superior discrimination compared with conventional regression approaches in clinical prediction. The small numerical difference between GBM and GLM therefore does not, by itself, justify the additional complexity of GBM, and logistic regression may remain preferable when simplicity and direct interpretability are prioritized. In the present study, SHAP was used to characterize the prediction patterns of the GBM rather than as a justification for choosing a more complex model over logistic regression. GBM was retained for subsequent explainability analysis and deployment because of its favorable overall discrimination and calibration profile and its capacity to model non-linear relationships, rather than because of a demonstrated AUC advantage.

Several of the key predictors identified in this study are consistent with established clinical experience in stroke care. Neurological deficit severity, as measured by the NIHSS, remained the strongest determinant of functional outcome, reflecting the direct impact of initial brain injury on subsequent recovery (27, 28). Age also showed a clear association with outcome, with higher risk observed at older ages, which is in line with reduced physiological reserve and recovery capacity (29, 30). Inflammatory status, reflected by C-reactive protein, contributed to outcome prediction in a non-linear manner, suggesting that inflammation may play a more prominent role beyond lower baseline levels (31, 32). Renal dysfunction was similarly associated with poorer outcomes, consistent with the broader vascular and metabolic burden carried by these patients (33, 34).

Not all predictors showed uniform patterns. Baseline LDL cholesterol demonstrated variable and context-dependent associations with outcome, which mirrors the inconsistent findings reported in previous studies (35, 36). This variability likely reflects the complex interplay between lipid metabolism, vascular disease, and treatment effects in acute ischemic stroke. Taken together, these observations suggest that meaningful prognostic information can be derived from a limited set of routinely available variables, without reliance on large or highly specialized feature sets.

The relative importance patterns observed across predictors were clinically intuitive. Measures of neurological severity primarily captured the extent of initial injury, whereas age reflected accumulated comorbidity and reduced recovery potential. The non-linear contribution of inflammatory markers supports the concept that systemic inflammation may act as a modifier of brain injury and repair rather than a simple linear risk factor. Reduced renal function may represent long-standing microvascular damage and impaired physiological resilience, which together limit tolerance to ischemic stress.

We also observed modest interaction effects between selected variables, particularly involving renal function, lipid levels, and smoking status. These interactions were not dominant drivers of model performance but suggest that vascular and metabolic factors may influence outcome differently across patient subgroups. Such patterns are difficult to capture with conventional regression approaches and highlight the potential value of flexible modeling frameworks when addressing heterogeneous clinical populations (37, 38).

From a practical perspective, several aspects of this work are relevant to routine stroke care. All predictors used in the model are available within the first 24 h of admission, allowing early risk assessment. A potential use of the model would be to identify patients with a higher predicted probability of poor 3-month functional outcome who may warrant closer prognostic assessment, early rehabilitation planning, or more structured follow-up. However, this use case was not evaluated in the present study, and model output should not be used to direct clinical management at this stage. Importantly, model explanations at the individual level may help clinicians contextualize predicted risks rather than rely solely on a single probability estimate. We did not compare the model with clinician judgment or established prognostic scores such as ASTRAL or iScore; therefore, its incremental clinical utility over current practice remains unknown. Accordingly, the contribution of this study lies in integrating prediction based on routine clinical data with multidimensional evaluation and patient-level interpretation, rather than in proposing a new algorithm or establishing superiority over existing prediction tools.

## Strengths and limitations

This study has several strengths. We focused on routinely collected clinical data, enhancing practical applicability. Feature selection was restricted to the training set to reduce overfitting, and model performance was evaluated using both discrimination and calibration metrics. The use of explainability analyses allowed closer examination of how individual predictors contributed to outcome estimates.

Several limitations should be acknowledged. This was a single-center retrospective study, and validation was limited to cross-validation and a held-out test set derived from the same source cohort. The held-out test set represented split-sample internal validation rather than external validation. No independent temporal or external cohort was available. Although these procedures assess internal reproducibility, they do not establish model transportability across institutions, geographic populations, time periods, or clinical workflows. An independent cohort containing the same early predictors and 3-month mRS outcome was not available for the present study; therefore, external or temporal validation could not be performed. The reported performance may consequently remain optimistic, and both the model and web calculator should be regarded as research prototypes until independently validated. The model was not compared with clinician judgment or established stroke prognostic scores, such as ASTRAL or iScore; therefore, its incremental value over current prognostic practice remains undetermined. Patients with active infection at admission were also excluded. This exclusion may limit the external validity of the model, particularly for AIS patients presenting with concurrent infection. Although 206 patients experienced poor 3-month functional outcome, the available sample size remained moderate for the development and comparison of multiple machine-learning algorithms. Training-set feature selection and cross-validation, together with evaluation in a held-out test set, were used to mitigate overfitting and assess internal performance. Nevertheless, the decrease in AUC from 0.910 in the training set to 0.872 in the test set, together with the corresponding decrease in AUCPR from 0.814 to 0.741, suggests some degree of model optimism and possible overfitting. This performance gap should be considered when interpreting the reported discrimination, particularly in the absence of independent external validation. Patients with unavailable 3-month mRS data were excluded, which may have introduced selection bias. TOAST classification was missing in 93 patients. LASSO feature selection therefore used training-set complete cases for the candidate predictors, whereas downstream H2O models retained patients with missing TOAST using native missing-value handling. Bias related to missing etiologic classification cannot be excluded. Imaging markers and treatment-related factors were not included and may provide additional prognostic information. Explainability analyses describe model behavior rather than causal mechanisms.

Future work should first externally validate the model in at least one independent cohort and subsequently assess its clinical impact, including whether its use improves clinical decision-making or patient outcomes beyond predictive performance alone.

## Conclusion

This study demonstrates that meaningful prediction of 3-month functional outcome after acute ischemic stroke can be achieved using a limited set of routinely collected clinical variables, while preserving interpretability at the individual patient level. By capturing non-linear effects and patient-specific variability, the proposed approach provides individualized risk estimates that warrant further clinical evaluation. Although external validation is required, the incremental value of the present model over current prognostic practice and its effect on clinical decision-making remain to be established before clinical implementation.

## Data Availability

The datasets generated and/or analyzed during the current study are not publicly available due to institutional data protection policies but are available from the corresponding author on reasonable request.
